# Characterization of a human placental clearance system to regulate serotonin levels in the fetoplacental unit

**DOI:** 10.1186/s12958-023-01128-z

**Published:** 2023-08-23

**Authors:** Frantisek Staud, Xin Pan, Rona Karahoda, Xiaojing Dong, Petr Kastner, Hana Horackova, Veronika Vachalova, Udo R. Markert, Cilia Abad

**Affiliations:** 1 Department of Pharmacology and Toxicology, Faculty of Pharmacy in Hradec Kralove, Charles University, Hradec Kralove, Czech Republic; 2 Placenta-Lab, Department of Obstetrics, Jena University Hospital, Jena, Germany; 3 Department of Obstetrics and Gynecology, The Second Affiliated Hospital of Chongqing Medical University, Chongqing, 400010 China; 4 Department of Pharmaceutical Chemistry and Drug Analysis, Faculty of Pharmacy in Hradec Kralove, Charles University, Hradec Kralove, Czech Republic

**Keywords:** Placenta, Serotonin, Fetal development, Homeostasis, Clearance

## Abstract

**Background:**

Serotonin (5-HT) is a biogenic monoamine with diverse functions in multiple human organs and tissues. During pregnancy, tightly regulated levels of 5-HT in the fetoplacental unit are critical for proper placental functions, fetal development, and programming. Despite being a non-neuronal organ, the placenta expresses a suite of homeostatic proteins, membrane transporters and metabolizing enzymes, to regulate monoamine levels. We hypothesized that placental 5-HT clearance is important for maintaining 5-HT levels in the fetoplacental unit. We therefore investigated placental 5-HT uptake from the umbilical circulation at physiological and supraphysiological levels as well as placental metabolism of 5-HT to 5-hydroxyindoleacetic acid (5-HIAA) and 5-HIAA efflux from trophoblast cells.

**Methods:**

We employed a systematic approach using advanced organ-, tissue-, and cellular-level models of the human placenta to investigate the transport and metabolism of 5-HT in the fetoplacental unit. Human placentas from uncomplicated term pregnancies were used for perfusion studies, culturing explants, and isolating primary trophoblast cells.

**Results:**

Using the dually perfused placenta, we observed a high and concentration-dependent placental extraction of 5-HT from the fetal circulation. Subsequently, within the placenta, 5-HT was metabolized to 5-hydroxyindoleacetic acid (5-HIAA), which was then unidirectionally excreted to the maternal circulation. In the explant cultures and primary trophoblast cells, we show concentration- and inhibitor-dependent 5-HT uptake and metabolism and subsequent 5-HIAA release into the media. Droplet digital PCR revealed that the dominant gene in all models was MAO-A, supporting the crucial role of 5-HT metabolism in placental 5-HT clearance.

**Conclusions:**

Taken together, we present transcriptional and functional evidence that the human placenta has an efficient 5-HT clearance system involving (1) removal of 5-HT from the fetal circulation by OCT3, (2) metabolism to 5-HIAA by MAO-A, and (3) selective 5-HIAA excretion to the maternal circulation via the MRP2 transporter. This synchronized mechanism is critical for regulating 5-HT in the fetoplacental unit; however, it can be compromised by external insults such as antidepressant drugs.

**Supplementary Information:**

The online version contains supplementary material available at 10.1186/s12958-023-01128-z.

## Background

The placenta is an ephemeral organ that was initially believed to only be important during the period of pregnancy. However, epidemiological studies have shown that *in utero* development is also essential for fetal programming of diseases later in life [1]. Optimal placental function is, therefore, essential beyond *in utero* life. Serotonin (5-hydroxytryptamine, 5-HT) is a biogenic monoamine with diverse functions in multiple human organs and tissues; it acts as a neurotransmitter in the central nervous system, a local mediator in the gut, and a vasoactive agent in the blood. Therefore, tightly regulated levels of this monoamine in the fetoplacental unit are critical for proper placental functions, fetal development, and programming.

Despite being a non-neuronal organ, the placenta expresses a suite of homeostatic proteins to regulate 5-HT levels. Specifically, it expresses functional membrane transporters for 5-HT uptake on both sides of the polarized trophoblast cells: a high-affinity but low-capacity 5-HT transporter (SERT/SLC6A4) on the maternal side [2] and a polyspecific low-affinity but high-capacity organic cation transporter (OCT3/SLC22A3) on the fetal side [3]. It has also been confirmed that the trophoblast expresses 5-HT-producing and 5-HT-degrading enzymes, namely tryptophan hydroxylase (TPH), which is the rate-limiting enzyme for 5-HT synthesis from maternally acquired tryptophan [4, 5], and monoamine oxidase A (MAO-A), which metabolizes 5-HT into 5-hydroxyindoleacetic acid (5-HIAA) [6, 7]. Numerous studies have attempted to describe 5-HT homeostasis in the placenta [4, 8, 9]; however, several questions remain unanswered. For example, 5-HT transport across the basal, fetus-facing membrane is still poorly understood. Similarly, the fate of 5-HIAA, the principal metabolite of 5-HT degradation, has never been investigated in the placenta.

Since 5-HIAA is not a ligand of 5-HT receptors, it has been considered an inert molecule with no functional activity. However, recent studies have indicated that it has multiple intracellular roles, including involvement in MAPK signaling, amyloid β clearance, or pain modulation [10–12]. It is, therefore, essential for cells producing 5-HIAA to regulate the homeostasis of this metabolite and control its excretion for detoxication purposes. In the brain and kidney, probenecid-dependent transporter(s) have been suggested to efflux 5-HIAA from the cells [13–16]. No such detoxification mechanism has yet been identified in the placenta, however.

In our previous research [3, 17–19], we used ex vivo approaches, cell-based experiments, and animal models to characterize monoamine homeostasis at the maternal-fetal interface. In the perfused rat placenta, we demonstrated that OCT3 effectively removes monoamines from fetal circulation for subsequent metabolism in trophoblast cells [3, 17]. In the present study, we hypothesize that a similar clearance mechanism might also be functionally expressed in the human placenta. Therefore, we used a series of human placenta models, including dually perfused human placenta cotyledon, placental explants, and isolated trophoblast cells to demonstrate that the human term placenta effectively clears 5-HT from the fetal circulation and actively excretes its metabolite, 5-HIAA, to the maternal circulation. We also demonstrate that this synchronized clearance system can be disrupted by external insults, such as antidepressant drugs.

## Methods

### Human placenta sample collection

Human term placentas were collected from uncomplicated pregnancies (gestation age at delivery: 38 to 40 weeks) delivered by caesarean sections at the University Hospital in Hradec Kralove (Czech Republic) or the University Hospital Jena (Germany). All experiments were performed in accordance with the Declaration of Helsinki, and human placenta samples were obtained with the women’s written informed consent. The study was approved by the University Hospital Research Ethics Committee (201,706 S17P) and the local Ethical Committee at the Friedrich-Schiller University Jena (038–02/03).

### Ex vivo perfusion of the human term placenta

Dual perfusion of the human placenta cotyledon was adapted in our laboratory [20] based on the method developed by Schneider et al. [21] and as described previously [22]. The maternal side was perfused in a closed system (perfusate recirculation) at a flow rate of 12 ml/min, and the fetal side was perfused in an open system at a flow rate of 3 ml/min. Several quality control parameters were measured to assess the accuracy and intactness of the perfusion system and the placental tissue integrity and viability. On the fetal side, the flow rate and intravascular pressure were monitored throughout the experiment. On the maternal side, the perfusate volume and human β-choriogonadotropin release were monitored. In both circuits (fetal and maternal), glucose and lactate concentrations and pH were measured. Further details on maternal and fetal perfusate composition and quality measurements are provided in the Supplementary methods.

5-HT was added to the fetal inflow compartment at two concentrations (100 nM and 100 µM) and samples were collected from the fetal outflow and maternal reservoir (see Supplementary Fig. 1). 5-HT and 5-HIAA levels were analyzed by HPLC, as described below. The placental capacity for removing 5-HT from the fetal circulation was expressed in terms of the extraction ratio (ER) [3].

### Human term placental explant isolation and culture

Human placental explants (n = 5) were used in follow-up experiments to investigate 5-HT homeostasis. Fragments of cotyledons were carefully separated by dissection from different areas of each placenta, and the chorionic plate and decidua were removed. Villous tissue was further dissected into explants of about 30 mg. Randomly sampled villous tissue sections of roughly 0.5 cm × 0.5 cm were cleaned of large vessels and blood clots and rinsed with cold sterile saline. Explants were then cultured in 12-well plates (TPP Techno Plastic Products AG, CH) in DMEM:F12 medium (with HEPES, glucose, and L-glutamine; Lonza Bioscience, USA) supplemented with 10% FBS, penicillin 100 U/ml, streptomycin 0.1 mg/ml, and amphotericin B 2.5 µg/ml [23] with 3 explants per well. The villous explants were incubated at 37 °C in an 8% O_2_, 5% CO_2_, 87% N_2_ atmosphere for 4 h to allow equilibration of the cultures and recovery from the isolation procedure. After the 4-hour incubation, the media was replaced, and explants were cultured for 24 h under normal conditions before the experiments began.

Explant viability was verified by two different methods: the incorporation of thiazolyl blue tetrazolium bromide (MTT) [24] and the release of the intracellular enzyme lactate dehydrogenase (LDH) into the incubation medium [25]. For the MTT assay, the explants were washed with Opti-MEM™ (Gibco, Thermo Fisher Scientific, USA) and incubated with 0.5 mg/ml MTT solution at 37 °C for 1 h. Subsequently, the explants were transferred to 1 ml DMSO and incubated for 5 min with shaking at room temperature. The supernatant’s absorbance (A) at 570 and 690 nm was then measured. Results are expressed as the difference between A570 and A690 per gram of tissue. LDH release into the incubation media was measured using the LDH Activity Assay Kit (Sigma Aldrich, USA), following the manufacturer’s instructions.

### Isolation of primary trophoblast cells from human term placenta

Primary trophoblast cells (n = 5) were isolated from the human term placenta to investigate the placental capacity to take up and metabolize 5-HT and excrete 5-HIAA at the cellular level. Villous tissue was subjected to enzymatic digestion at 37 °C with 0.25% trypsin (Gibco, Thermo Fisher Scientific, USA) and 300 IU/mL deoxyribonuclease I (Sigma-Aldrich, USA) according to the protocol of Kliman et al. with minor modifications [26, 27]. The cell suspension was collected in Dulbecco’s Modified Eagle Medium (DMEM; high glucose, GlutaMAX™; Thermo Fisher Scientific, USA) and overlaid on a discontinuous Percoll^®^ gradient (Sigma-Aldrich, USA). Percoll gradient-purified trophoblasts were seeded in Nunc™ MicroWell™ 96-Well plates (Delta-Treated; Thermo Fisher Scientific, USA) and cultured in DMEM (high glucose, GlutaMAX™) supplemented with 10% FBS, penicillin 100 U/ml, and streptomycin 0.1 mg/ml at 37 °C, 5% CO_2_, 95% N_2_, replacing the medium daily.

To determine the purity of isolated cells, flow cytometry was used to quantify the percentage of cells stained with directly-labeled antibodies for cytokeratin 7 (epithelial cells - positive for primary trophoblast cells), vimentin (mesenchymal cells, fibroblasts, stromal cells), and Von Willebrand factor (endothelial cells) [27]. All antibodies were obtained from Novus Biologicals, USA. At least 10,000 cells were analyzed using a SP6800 Spectral Cell Analyzer (Sony Biotechnology, USA) and data analysis was performed using FCS Express Software 7.0 (De Novo Software, USA). Purity results are shown in Supplementary Table 1.

### 5-HT metabolism and excretion in placental explants and trophoblast cells

Functional experiments were performed 72 h after primary cell isolation, representing the differentiated syncytiotrophoblast stage, and 40 h after placental explant isolation. Culturing media was removed, and the cells/explants were treated with medium supplemented with either 100 nM or 100 µM 5-HT in the absence/presence of different inhibitors for 1, 2, 4, and 6 h. The following inhibitors were used: phenelzine (MAO-A inhibitor, 100 µM) [28], paroxetine (SERT and OCT3 inhibitor, 100 µM) [3], probenecid (non-specific inhibitor of organic anion transporters, 500 µM) [29], and MK-571 (selective inhibitor of MRP2, 50 µM) [30]. At the end of the incubation period the cell medium was collected and centrifuged at 1,500x *g* for 15 min at 4 °C. The cell-free supernatant was then stored at -80 °C until HPLC measurements, as described below. The results are expressed as the ratio of the 5-HIAA and 5-HT concentrations. The experimental process is depicted schematically in Supplementary Fig. 1.

### HPLC analysis of 5-HT and 5-HIAA

Concentrations of 5-HT and 5-HIAA in the cell-free supernatant and placental perfusate were determined using a Shimadzu LC20 Performance HPLC chromatograph (Shimadzu, JP) as described previously [3]. A Kinetex EVO C18 100 A 150 × 3 mm, particle size 5 μm column (Phenomenex, USA) with a guard column was used. Analytes were eluted with a biphasic mobile phase consisting of A − 3:97 (v/v) methanol:acetic acid (0.1 M, pH 4.5, adjusted with NaOH) and B - methanol. The proportion of B was 0% for 0–8.4 min, then linearly increased to 20% at minute 9.6, held at 20% until minute 22.6, linearly decreased to 0% after minute 23.2 then held at 0% until minute 30. Excitation and emission wavelengths of the fluorescence detector were set for individual compounds: 280/334 nm for 5-HT from 0 to 6.5 min and 276/333 nm for 5-HIAA from 6.5 to 18 min.

### Absolute quantification of ***SERT, OCT3, MAO-A***, and ***MRP2*** transcripts

RNA was isolated from the tissue/cells using Tri Reagent (Molecular Research Center, USA), according to the manufacturer’s instructions. RNA purity and concentration were measured using a NanoDrop™ 1000 Spectrophotometer (Thermo Fisher Scientific, USA). Reverse transcription was performed using the iScript Advanced cDNA Synthesis Kit (BioRad, USA), following the manufacturer’s protocol. Subsequently, the obtained cDNA (25 ng/µl) was mixed with the ddPCR™ Supermix for Probes (BioRad, USA) and predesigned probes (always in a duplex setup with FAM probes for target genes and HEX probe for reference gene). The following target genes were analyzed: *SERT* (Hs00984349_m1), *OCT3* (Hs01009571_m1), *MAO-A* (Hs00165140_m1), and *MRP2* (Hs00960489_m1); all obtained from Thermo Fisher Scientific. Beta-2-Microglobulin (*B2M*) was used as the reference gene (dHsaCPE5053101; BioRad, USA). Droplets were generated using the QX200 Droplet Generator and amplified to the end-point using the T100™ Thermal Cycler as previously described [3]. Droplet reading was performed on a QX200™ Droplet Reader and target gene concentration was calculated using QuantaSoft™ Software. The threshold droplet count for final data evaluation was set to 13 000. The results are shown as transcripts/ng of transcribed RNA. All reagents, consumables, and equipment for ddPCR analysis were obtained from BioRad unless otherwise stated.

### Statistical analysis

Data analysis and graphical presentation were performed in GraphPad Prism version 9.2.0. Because we observed high interindividual variability between samples, results for individual samples are plotted with lines connecting median values. Statistical significance was evaluated using a Mixed-effects model with Geisser-Greenhouse correction and Dunnett’s multiple comparisons test, the Mann-Whitney test, or one-way ANOVA. *p ≤ 0.05, **p ≤ 0.01, ***p ≤ 0.001.

## Results

### Placental perfusion parameters

**Fig. 1:**
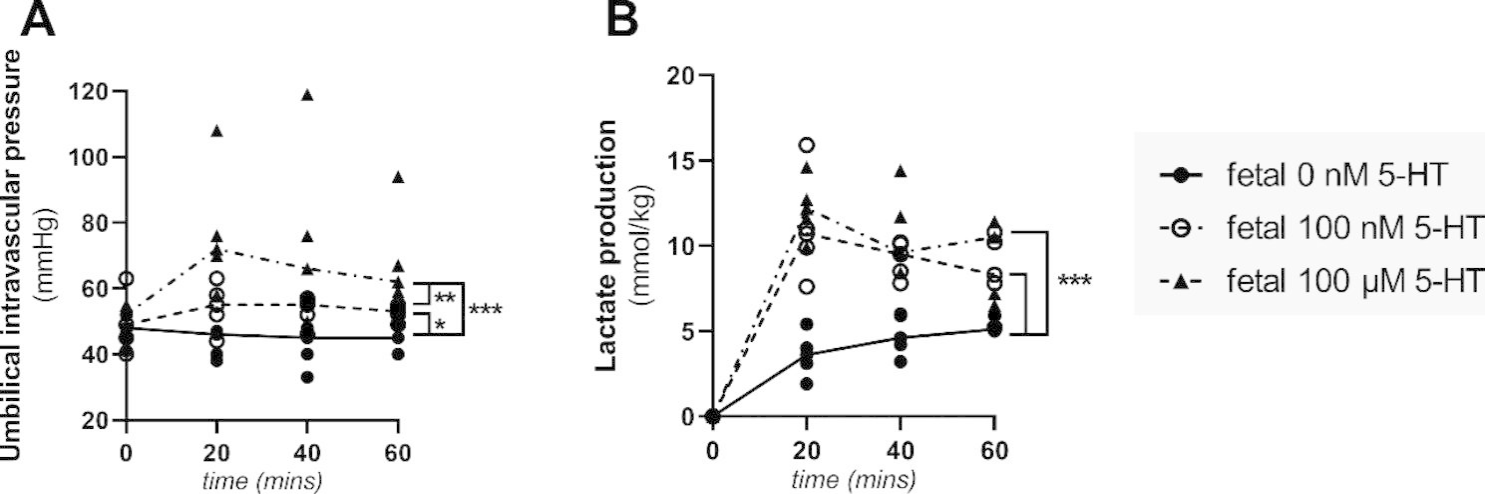
Effect of 5-HT concentration on umbilical intravascular pressure and lactate production. Adding 5-HT to the fetal circulation increased the intravascular pressure in the umbilical arteries (**A**) and lactate production in the fetal circuit (**B**). Data are presented for individual biological replicates with lines connecting median values; n = 5. Statistical significance was evaluated using a mixed effects model with the Geisser-Greenhouse correction and Dunnett’s multiple comparisons test. * p ≤ 0.05, ** p ≤ 0.01, *** p ≤ 0.001

To determine the transfer and distribution of fetal 5-HT in the human placenta, experiments were performed on isolated cotyledons using a dual side perfusion system with the maternal circuit being closed and the fetal side being open. The fetal side of the placenta was perfused with solutions containing 5-HT at concentrations of 0 nM, 100 nM, or 100 µM. Several parameters were monitored for quality control purposes and to ensure the validity of the perfusion experiments. Tissue integrity and viability was confirmed by evaluating the fetal flow rate, placental metabolism (glucose consumption, lactate production, and β-hCG release), and pH values in the maternal and fetal circuits [22]. The metabolic activity results were normalized to the weight of the cotyledon. Throughout the experiment, the tissue maintained continuous glucose consumption, lactate production, β-hCG release and stable pH (Supplementary Fig. 2). However, upon raising the 5-HT concentration in the fetal circulation from 0 nM to 100 nM or 100 µM during the perfusion stage, both fetal intravascular pressure (Fig. 1A) and lactate production (Fig. 1B) increased.

### Disposition of 5-HT and 5-HIAA in the perfused human term placenta

**Fig. 2:**
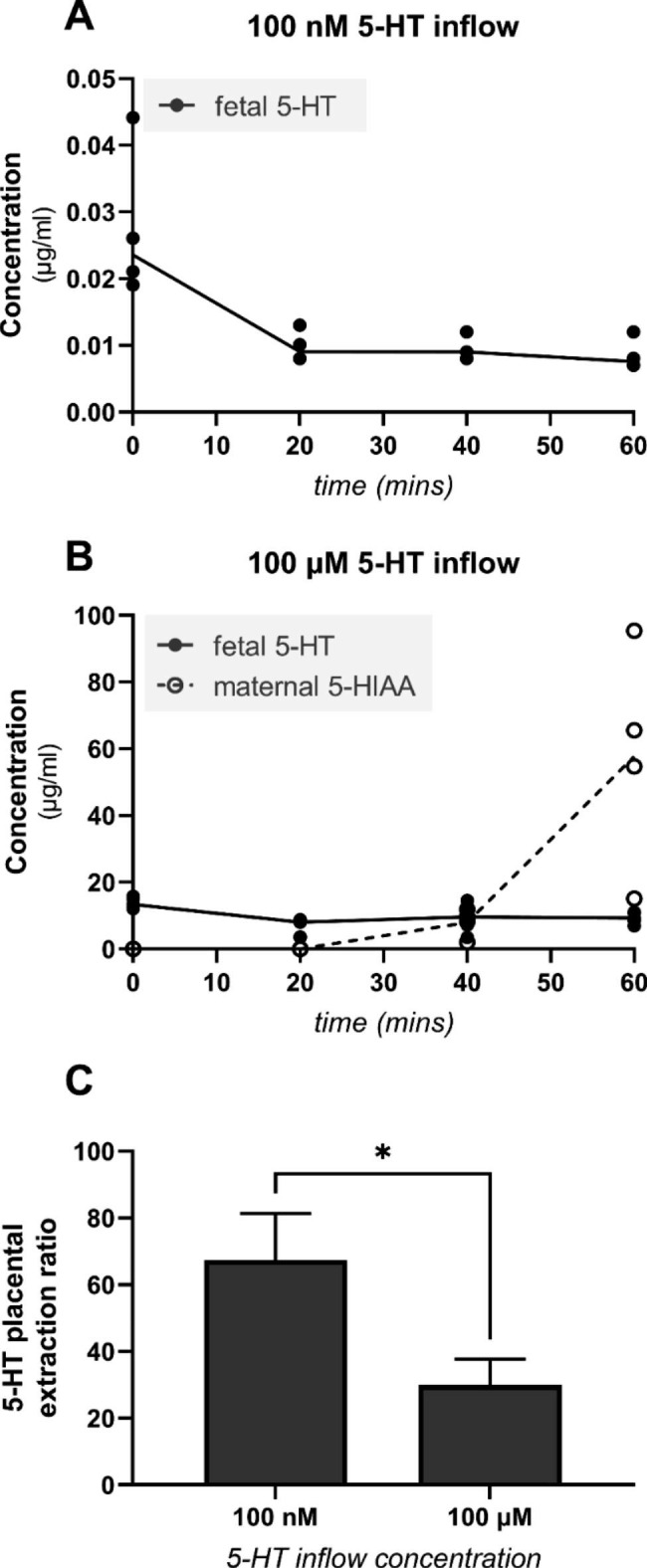
Placental uptake and metabolism of fetal 5-HT in the dually perfused human placenta. 100 nM (**A**) or 100 µM (**B**) 5-HT was added to the fetal inflow compartment and samples were collected from the fetal outflow and maternal reservoir for analysis of 5-HT and 5-HIAA levels. At a fetal inflow 5-HT concentration of 100 nM, no 5-HIAA was detected in the maternal reservoir or fetal outflow (**A**). However, after 20 min of perfusion with 100 µM 5-HT, 5-HIAA was detected in the maternal reservoir (**B**). Additionally, the placental capacity to remove 5-HT from the fetal circulation was concentration-dependent (**C**). Data are presented for individual biological replicates with lines connecting median values or as medians with interquartile ranges; n = 5. Statistical significance was evaluated using the Mann-Whitney test. * p ≤ 0.05

To evaluate the placental uptake and metabolism of fetal 5-HT, samples were collected from the fetal outflow and maternal reservoir for analysis of 5-HT and 5-HIAA levels. The 5-HT inflow concentration strongly affected the placental capacity to remove 5-HT from the fetal circulation: at a physiological (100 nM) inflow concentration, the placenta extracted a median of 67.37% of the fetal side 5-HT during a single pass through the organ, but at supraphysiological 5-HT concentrations (100 µM) its capacity fell to 29.96% (Fig. 2C). At the lower inflow concentration (100 nM), 5-HT was detected only in the fetal outflow and no 5-HIAA was quantified in either circuit (Fig. 2A). However, at the higher 5-HT inflow concentration (100 µM), 5-HIAA was detected in the maternal circuit after 20 min of perfusion and its concentration in the maternal circuit increased substantially as the perfusion experiment proceeded (Fig. 2B). No 5-HIAA was quantified in the fetal outflow.

These results suggest the existence of a placental clearance mechanism for fetal 5-HT involving saturable transporter-mediated uptake of 5-HT from the fetal circulation, its conversion to 5-HIAA within the placenta, and exclusive excretion of the metabolite towards the maternal compartment.

### 5-HT clearance in human placental explants and primary trophoblast cells

To address 5-HT metabolism within the placenta, and the efflux of 5-HIAA to the maternal circulation, we conducted experiments in placental explant cultures and primary trophoblast cells isolated from human term placenta. 5-HT was added to the explants/primary cells at concentrations of 100 nM or 100 µM and the cell-free supernatant was analyzed for 5-HT and 5-HIAA for 1, 2, 4, and 6 h. To verify tissue viability during explant experiments and under different experimental conditions, the culture media was analyzed for LDH activity, and the tissue was examined for MTT incorporation. Both analyses confirmed that the tissue remained viable for up to 6 h (Supplementary Fig. 3).

**Fig. 3:**
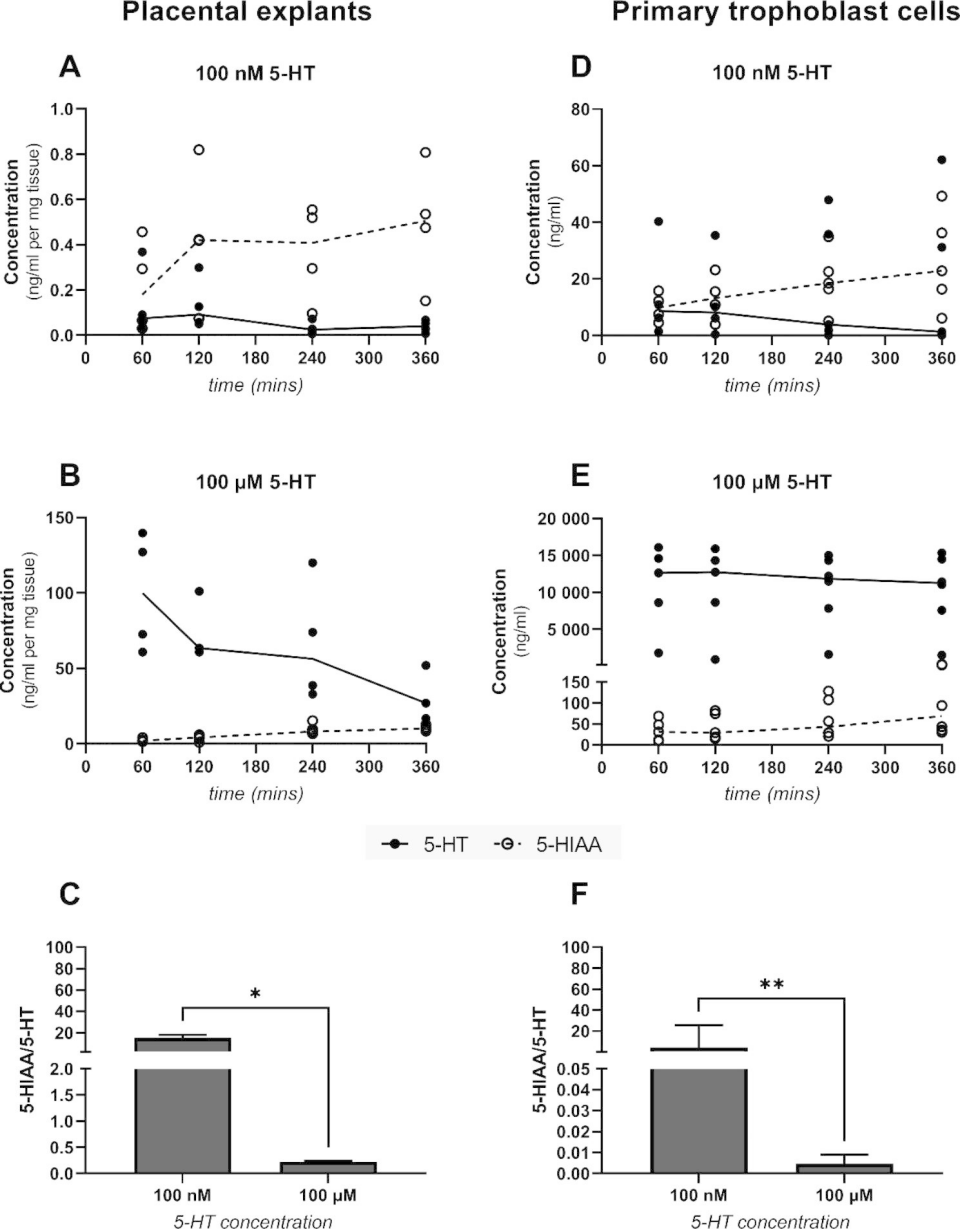
5-HT clearance by isolated placental explants and primary trophoblast cells. The capacity of tissue samples and cells to take up 5-HT and metabolize it to 5-HIAA was evaluated at 5-HT concentrations of 100 nM (**A, D**) and 100 µM (**B, E**) over a period of 6 h. This process was concentration-dependent, with the 100 nM substrate concentration giving significantly higher 5-HIAA/5-HT ratios at 4-hour incubation time (**C, F**). Data are presented for individual biological replicates with lines connecting median values or as medians with interquartile ranges; n = 5. Statistical significance was evaluated using the Mann-Whitney test. * p ≤ 0.05, ** p ≤ 0.01

In both models, the 5-HT concentration in the culture media decreased and 5-HIAA levels increased over time (Fig. 3A, B, D and E). At the lower 5-HT concentration, 5-HIAA levels exceeded those of 5-HT (Fig. 3A and D), while the opposite was observed at the higher 5-HT concentration (Fig. 3B and E). This concentration-dependent effect on 5-HT metabolism is also evidenced by the 5-HIAA/5-HT ratios, which were approximately 70-fold and 900-fold higher at the low 5-HT concentration than at the high 5-HT concentration in placental explants and primary cells, respectively (Fig. 3C F).

### Effect of inhibitors on 5-HT clearance by placental explants and primary trophoblast cells

**Fig. 4:**
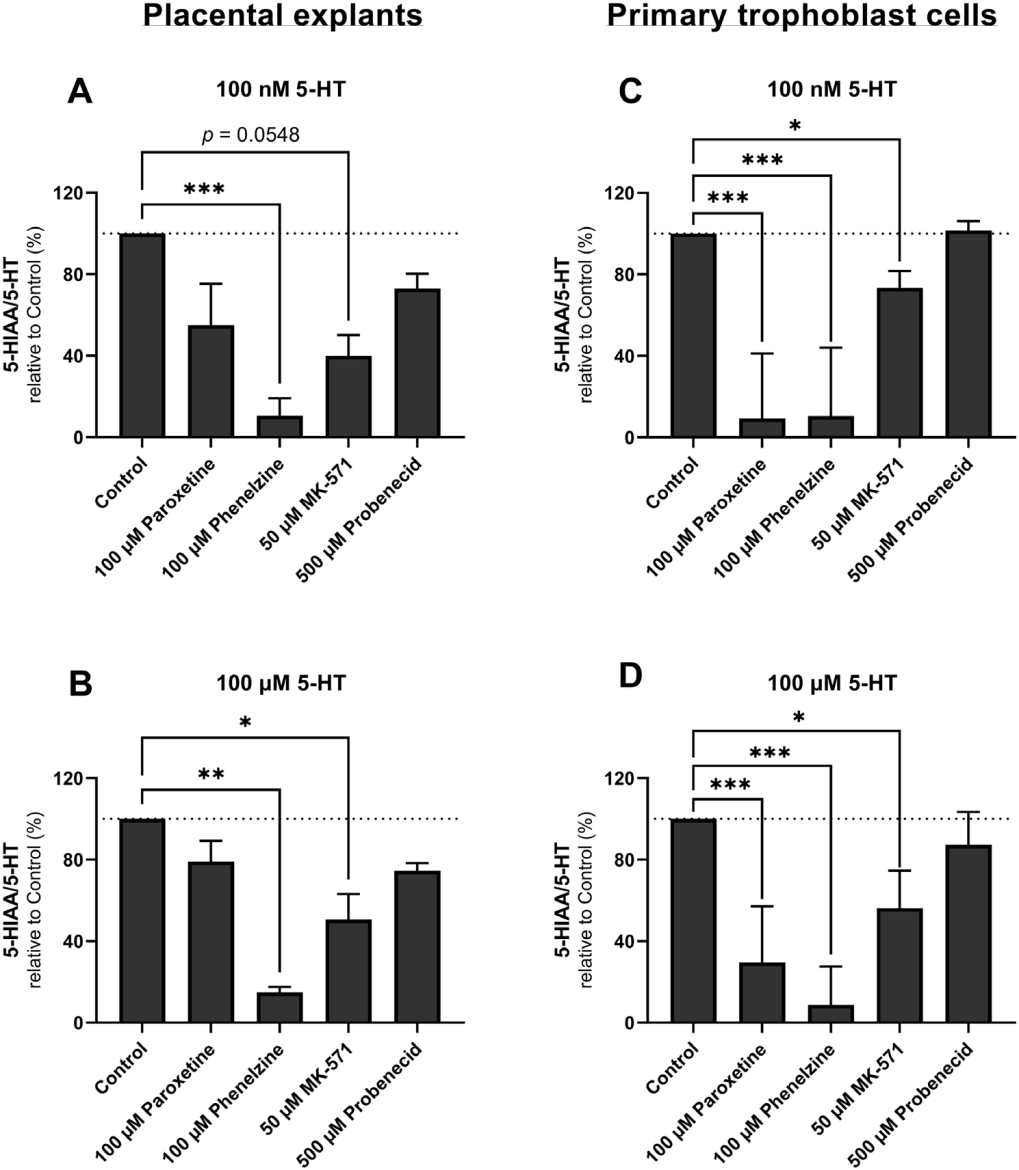
Effect of selected inhibitors on 5-HT clearance by placental explants (**A, B**) and primary trophoblast cells (**C, D**). The uptake of 5-HT and release of 5-HIAA was measured at initial 5-HT concentrations of 100 nM (**A, C**) and 100 µM (**B, D**) in the presence of 100 µM paroxetine (SERT and OCT3 inhibitor), 100 µM phenelzine (MAO-A inhibitor), 50 µM MK-571 (MRP2 inhibitor), or 500 µM probenecid (OAT inhibitor). The extent of 5-HT metabolism over a period of 4 h is expressed as the ratio of the concentrations of 5-HIAA and 5-HT. The results are shown relative to the control condition (CTRL) with no added inhibitor. Data are presented as medians with interquartile ranges; n = 5. Statistical significance was evaluated using the Mann-Whitney test. * p ≤ 0.05, ** p ≤ 0.01, *** p ≤ 0.001

To investigate the mechanisms mediating placental clearance of 5-HT, we performed 4-hour incubations of placental explants and trophoblast cells with 5-HT and inhibitors targeting selected steps of 5-HT homeostasis, namely 5-HT uptake, 5-HT metabolism to 5-HIAA, and 5-HIAA efflux. At both 5-HT concentrations, the 5-HIAA/5-HT ratio was significantly reduced in the presence of phenelzine in both placental models (Fig. 4). Additionally, MK-571 treatment significantly reduced the 5-HIAA/5-HT ratio in primary trophoblast cells at both 5-HT concentrations (Fig. 4C and D). In placental tissue explants, MK-571 significantly reduced the 5-HIAA/5-HT ratio when the 5-HT concentration was 100 µM and had an almost significant effect (p = 0.0548) when the 5-HT concentration was 100 nM. Because MK-571 inhibits the MRP2 transporter, these results indicate that this transporter is involved in 5-HIAA efflux.

Collectively, we show that in the complex three-dimensional explant villous structure, 5-HT metabolism and 5-HIAA efflux are the dominant processes affected by external insults (phenelzine and MK-571, respectively). On the other hand, at the cellular level, trophoblast 5-HT clearance was successfully blocked at all three metabolic steps: 5-HT uptake by SERT and OCT3 (inhibited by paroxetine), metabolism by MAO-A (inhibited by phenelzine), and 5-HIAA excretion by MRP2 (inhibited by MK-571).

### Gene expression analysis of ***SERT***, ***OCT3***, ***MAO-A***, and ***MRP2*** by ddPCR

**Fig. 5:**
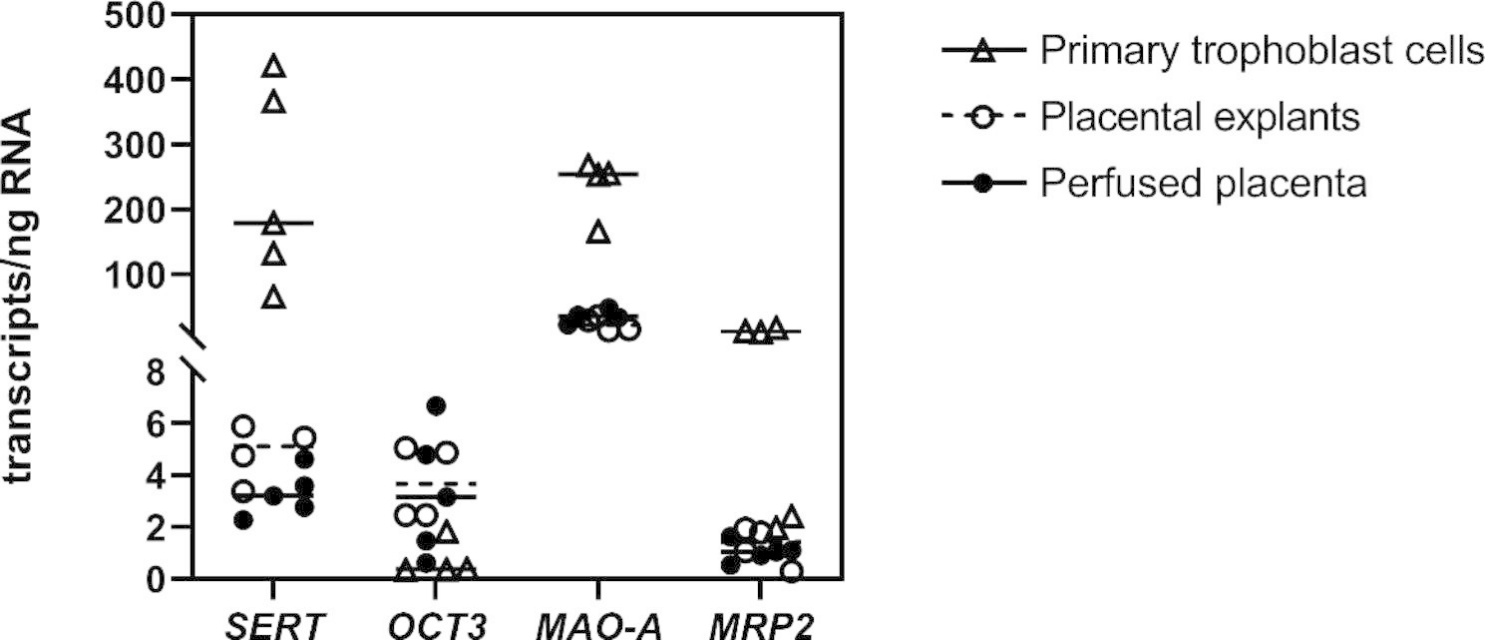
Absolute transcript quantification of key placental components involved in placental 5-HT clearance using digital droplet PCR analysis. Expression of *SERT*, *OCT3*, *MAO-A*, and *MRP2* was analyzed in the placental tissues used in the perfusion and explant studies, and in primary trophoblast cells isolated from human term placenta. The results are reported in numbers of transcripts per ng of transcribed RNA. Data are presented as individual biological replicates with horizontal lines indicating median values; n = 5

The ddPCR method was employed to analyze the transcript numbers of the main transporters and enzymes in the placental tissue (from perfusion and explant studies) and primary trophoblast cells. In general, primary trophoblast cells had the highest transcript numbers for all of the studied genes (Fig. 5), which we attribute to the purity of the isolated trophoblast cells and the lack of contamination by other placental cell types. The dominant gene in all models was *MAO-A*, supporting the crucial role of 5-HT metabolism in placental 5-HT clearance. *SERT*, *OCT3*, and *MRP2* had similar transcript numbers in the tissue models (< 10 transcripts/ng RNA).

## Discussion

**Fig. 6:**
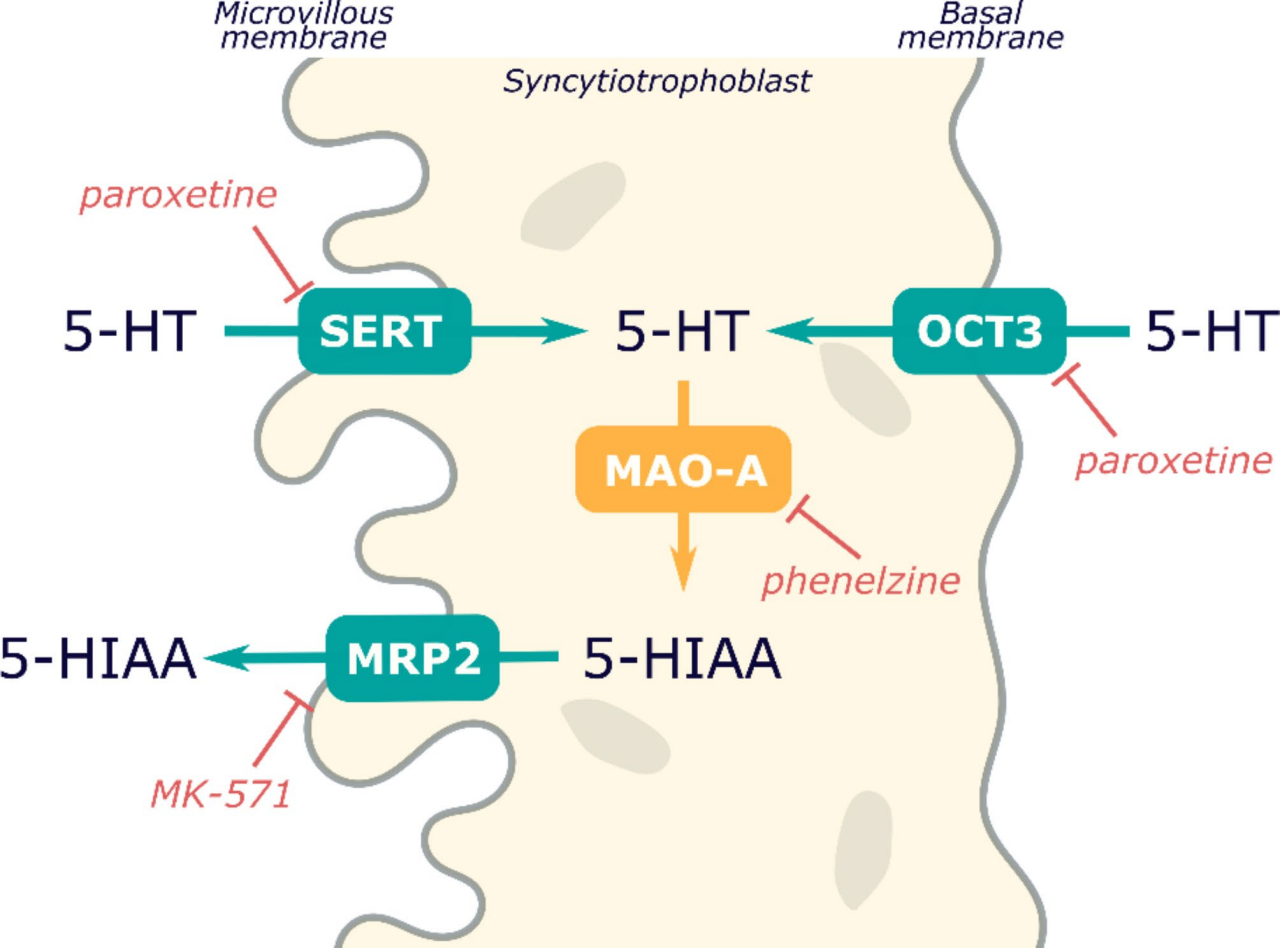
Schematic depiction of the human placenta clearance system of fetal 5-HT.

Strictly regulated homeostasis of 5-HT in the fetoplacental unit during gestation is crucial for proper placental and fetal development. Any disruption of this system, resulting in hyper- or hypo- serotonemia, is linked with poor pregnancy outcomes [31, 32]. Here, we used a systematic approach based on advanced human placenta models to provide novel molecular evidence of an efficient 5-HT clearance system within the human placenta whereby 5-HT is removed from the fetal circulation by membrane transporters and then metabolized into 5-HIAA inside the trophoblast cells by MAO-A (Fig. 6).

Using a dually perfused human placenta model system, we showed that up to 67% of 5-HT was removed from the fetal circulation during a single passage through the placenta at low physiological concentrations; this value dropped to 29% at high supraphysiological 5-HT concentrations, suggesting a saturable transport system. These results are consistent with those observed in the perfused rat placenta [3]. In many organs, including the placenta, 5-HT is deactivated by MAO-A-mediated metabolism to 5-HIAA; however, the fate of this metabolite in the placenta has never been investigated. Surprisingly, when its concentration was measured in the maternal and fetal circulations, detectable quantities were only found in the maternal circulation. This is consistent with the reported lack of 5-HIAA in cord blood [33]. In addition, using a closed maternal and fetal placental perfusion system, Mathiesen et al. showed a preferential secretion of 5-HIAA to the maternal circulation over 6 h of perfusion [34]. Altogether, these results suggest an efficient placental uptake of 5-HT from the fetal circulation followed by metabolism to 5-HIAA and predominant excretion to the maternal circulation.

Placental metabolism of 5-HT and production of 5-HIAA have only been reported in whole-tissue homogenates [7, 29, 35], which cannot be used to study the metabolite’s subsequent cellular disposition and membrane transport. Therefore, to investigate the mechanisms responsible for 5-HT uptake and 5-HIAA efflux by the trophoblast cells in more detail, we conducted experiments using placental explant cultures and primary trophoblast cells. Placental explants have the advantage of retaining the original tissue’s microstructure but cannot be used to study the activities of individual placental cell types [36]. Primary trophoblast cells are therefore a valuable complementary model system that can be used to study functions at the cellular level. Here, we show for the first time that human trophoblast cells and placental explants efficiently take up 5-HT and metabolize it to 5-HIAA. This was evidenced by steady reductions in the concentration of 5-HT and increases in that of 5-HIAA in the cell-free supernatant over time. To quantify the placenta’s capacity to clear 5-HT, we measured the ratio of the concentrations of the metabolite and substrate (5-HIAA/5-HT). In both models, we found that the 5-HIAA/5-HT ratio was concentration-dependent, confirming that the process is saturable. Moreover, this ratio was significantly reduced by inhibiting 5-HT transport and metabolism (Fig. 4.).

We further hypothesized that excretion of 5-HIAA from the trophoblast cells is vital for detoxication, so a functional efflux mechanism must exist in the cell membrane of the trophoblast cells. Since 5-HIAA is an organic acid, it cannot cross biological membranes by passive diffusion; instead, it must exit the cells via an efflux membrane transport system. Active mechanisms of 5-HIAA excretion in the human and rat brain and kidney were reported to be probenecid-dependent [13–16], indicating the involvement of an organic anion transporter (OAT) [35] and/or multidrug resistance-associated protein 2 (MRP2) [37]. To test this hypothesis, we investigated the effects of the organic anion transporter inhibitor, probenecid, and the MRP2 inhibitor, MK-571, on 5-HT clearance in placental explants and trophoblast cells. In both models, MK-571 had a statistically significant effect on the 5-HIAA/5-HT ratio, suggesting that MRP2 is involved in 5-HIAA extraction from the placenta. Our conclusions are supported by the fact that in the placenta, MRP2 is expressed exclusively in the mother-facing apical membrane [38]. Therefore, this ATP-driven efflux transporter is probably responsible for the active efflux of 5-HIAA from the trophoblast cells to the maternal circulation. Although long regarded as an inactive 5-HT metabolite, 5-HIAA was recently shown to have significant intracellular activity; it can negatively regulate RAS/MAPK signaling [12], which is responsible for modulating a wide range of cellular events including differentiation, proliferation, and cell death as well as eliciting inflammatory response [39]. We therefore speculate that MRP2-mediated 5-HIAA efflux from the placenta is the final critical component of the optimal placental 5-HT clearance system. Although not investigated in this study, recent literature indicates that after efflux of 5-HIAA to the maternal blood, it is removed from the body by renal excretion [40]. Importantly, MRP2 expression in the human placenta has been shown to increase during gestation, with peak expression at term [41]. This expression pattern parallels that of the other 5-HT detoxifying proteins (SERT, OCT3, and MAO-A) [27, 42] and further supports the hypothesis that placental 5-HT uptake and degradation increase progressively until term.

Notably, both primary cells and explants were highly sensitive to the MAO-A inhibitor phenelzine, highlighting this enzyme’s key role in placental 5-HT homeostasis. This dominant role of MAO-A in the “5-HT detoxication” system is supported by transcriptional data: MAO-A transcripts significantly outnumber those of SERT, OCT3, and MRP2 in all placental models. The importance of this enzyme during *in utero* development was recently highlighted in a longitudinal study that identified placental MAO-A as a biological mediator in the relationship between prenatal stress and infant temperament [43]. Importantly, reduced MAO-A expression has been observed in placentas from preeclamptic pregnancies [7, 44].

Taken together, here we present transcriptional and functional evidence that the human placenta has an efficient 5-HT clearance system involving (1) removal of 5-HT from the fetal circulation by OCT3, (2) metabolism to 5-HIAA by MAO-A, and (3) selective 5-HIAA excretion to the maternal circulation via the MRP2 transporter. This synchronized mechanism is critical for regulating 5-HT homeostasis in the fetoplacental unit. However, it is reasonable to assume that a perturbation of any of these components could dysregulate 5-HT levels in the fetoplacental unit. For example, antidepressants, mainly from the group of selective serotonin reuptake inhibitors (SSRIs) and serotonin and noradrenaline reuptake inhibitors (SNRIs), are reportedly prescribed to 13% of pregnant women [45], and their use has been linked to poor pregnancy outcomes [46]. These drugs inhibit both SERT and OCT3 transporters [47] and, therefore, can block serotonin uptake by trophoblast cells across both apical and fetal membranes [48]. In our study, we used paroxetine as an antidepressant with the strongest inhibitory effect to both SERT and OCT3 [48], and phenelzine as a model MAO-A inhibitor. We observed a statistically significant effect of both drugs on 5-HT clearance by placental explants and primary trophoblast cells (Fig. 4). In addition, metformin, often used in gestational diabetes mellitus, is a recognized substrate and inhibitor of organic cation transporters [49] including OCT3 in the placenta [50]. Notably, endogenous cortisol is an inhibitor of OCT3, and we have recently demonstrated its effect on OCT3-mediated uptake of 5-HT ex vivo and in situ [3]. We can therefore speculate that long-term prenatal use of antidepressants, metformin, or increased cortisol levels (due to chronic stress) may disrupt 5-HT levels in the fetoplacental unit and affect pregnancy outcomes.

A key strength of our study is its use of complementary models based on the human placenta ranging from perfused organs to isolated trophoblast cells. These models have been evaluated and used in several studies to investigate placental physiology, pathology, and pharmacology. The study’s main limitation is that only term placenta models were used, so the results provide no insight into earlier stages of gestation. Given the dynamic changes in the placenta during pregnancy, including those involved in 5-HT disposition [27, 42] it would be interesting and important to evaluate the placental capacity to clear fetal 5-HT at earlier stages of gestation. Furthermore, our inhibitory studies show only acute effects of paroxetine on placental clearance of 5-HT. Long-term studies using clinically relevant inhibitors are needed to identify their chronic effects on placental 5-HT homeostasis; investigations along these lines are currently underway in our lab. Further research should focus on using recently developed sequencing and metabolomic approaches to determine the influence of placental 5-HT on health and disease in the early stages of pregnancy. Moreover, identifying the effects of epigenetic and environmental impacts on this system could reveal opportunities to develop diagnosis and intervention strategies to prevent pregnancy-related diseases.

## Conclusions

In summary, we have shown for the first time that the human term placenta extracts 5-HT from the fetal circulation via OCT3-mediated transport and metabolizes it to 5-HIAA via trophoblast-located MAO-A. This metabolite is then unidirectionally excreted by MRP2 to the maternal circulation exclusively. The synchronized activity of these two membrane uptake transporters and the cytosolic degradation enzyme MAO-A allows the placenta to regulate 5-HT and 5-HIAA levels in the fetoplacental unit. In addition, given the vasoconstrictive action of 5-HT in umbilical arteries, we hypothesize that this clearance mechanism protects against local hyperserotonemia in the placental circulation. This clearance system could be of vital importance, as several studies have suggested that disrupted placental 5-HT homeostasis is involved in the etiology of preeclampsia [7, 44, 51, 52]. This resembles the situation in the lungs, where 5-HT is also actively removed from the circulation by a membrane transporter and metabolized by MAO-A; disruption of this clearance system has been linked to pulmonary hypertension [53, 54]. We propose that all steps of this delicate detoxication process can be affected by external insults, including prenatal antidepressants, which may lead to inefficient placental clearance of 5-HT and thereby jeopardize placental/fetal development.

### Electronic supplementary material

Below is the link to the electronic supplementary material.

**Additional file 1**: Supplementary methods (Figures S1-S3, Table S1)

## Data Availability

All data generated or analysed during this study are included in this published article (and its additional files).

## Abbreviations

5-HIAA: 5-hydroxyindoleacetic acid
5-HT: Serotonin
DMEM: Dulbecco’s Modified Eagle Medium
ER: Extraction ratio
FBS: Fetal bovine serum
HPLC: High-performance liquid chromatography
LDH: Lactate dehydrogenase
MAO-A: Monoamine oxidase-A
MRP2: Multidrug resistance-associated protein 2
MTT: Thiazolyl blue tetrazolium bromide
OCT3: Organic cation transporter 3
PCR: Polymerase Chain Reaction
SERT: Serotonin transporter
TPH: Tryptophan hydroxylase
