# Supplementary material for: Characterization of a human placental clearance system to regulate serotonin levels in the fetoplacental unit
Source: Reprod Biol Endocrinol. 2023 Aug 23;21:74. doi: 10.1186/s12958-023-01128-z (PMC10464227; doi:10.1186/s12958-023-01128-z)
Supplement: Supplementary file 1 — Additional file 1: Supplementary methods (Figures S1-S3, Table S1) [file 12958_2023_1128_MOESM1_ESM.docx]

**SUPPLEMENTARY MATERIALS**

**Human term placenta perfusion - perfusate composition and quality measurements**

The maternal perfusion solution consisted of 1.0 l (66.7%) NCTC-135-Medium with L-Glutamine (AppliChem, Germany), 0.5 l (33.3%) Earl's buffer, 12.0 g (8.0 g/l) bovine serum albumin (BSA) fraction V (Carl Roth, Germany), 3.0 g (2.0 g/l) Dextran FP 40 (SERVA electrophoresis, Germany), 2.0 g (1.33 g/l) D-Glucose (Merck, Germany), and 0.5 ml (0.33 ml/l) Heparin (Heparin-Rotexmedica 25000 I.E./5 ml, Rotexmedica, Germany). The fetal perfusion solution was supplemented with 48.0 g (32.0 g/l) BSA fraction V and 12.0 g (8.0 g/l) Dextran FP 40. Both solutions were adjusted to pH 7.4 with 1 M NaOH and sterile filtered through a 0.2 μm pore membrane (Thermo Fisher Scientific, Germany). Before entering the placenta, the medium was supplemented with 95% O_2_/5% CO_2_ in the maternal circuit and 95% N_2_/5% CO_2_ in the fetal circuit by using gas exchange oxygenators (Living Systems Instrumentation, USA). For valid perfusions, the allowed tolerance of fetal flow rate deviation was maximally 10% (3.0 ± 0.3 ml/min). For each experiment, 400 ml of perfusion solution, warmed-up to 37°C, was used to remove blood from the villous vascular compartment and the intervillous space for 30 min. For the 3 h experimental phase, 150 ml maternal perfusion solution was pumped with a flow rate of 12 ml/min through the closed maternal circuit. At pre-dose (0 min) and at 30-, 60-, 90-, 120-, 150-, and 180-min post-dose, samples from maternal circuit were collected. For the fetal circuit, samples were collected at pre-dose (0 min) and at 20-, 40-, and 60-min post-dose in media with different 5-HT concentrations. Samples were centrifuged (380x *g*, 10 min and stored at -80°C until respective analyses.

The fetal pressure was monitored continuously over the entire perfusion period by using a pressure control device, constructed by the Technical Service Department of the Jena University Hospital. Total maternal ß-hCG concentrations were assessed by applying an Architect Chemiluminescent Microparticle Immunoassay (CMIA) from Abbott (7K78, Institute of Clinical Chemistry and Laboratory Medicine, Jena University Hospital). Glucose and lactate concentrations as well as pH were determined by a blood gas analyzer ABL825 flex (Radiometer, Germany). When irregularities of quality parameters occurred (mostly in the fetal flow rate or pressure), the perfusion was considered invalid, terminated, and excluded from the study.


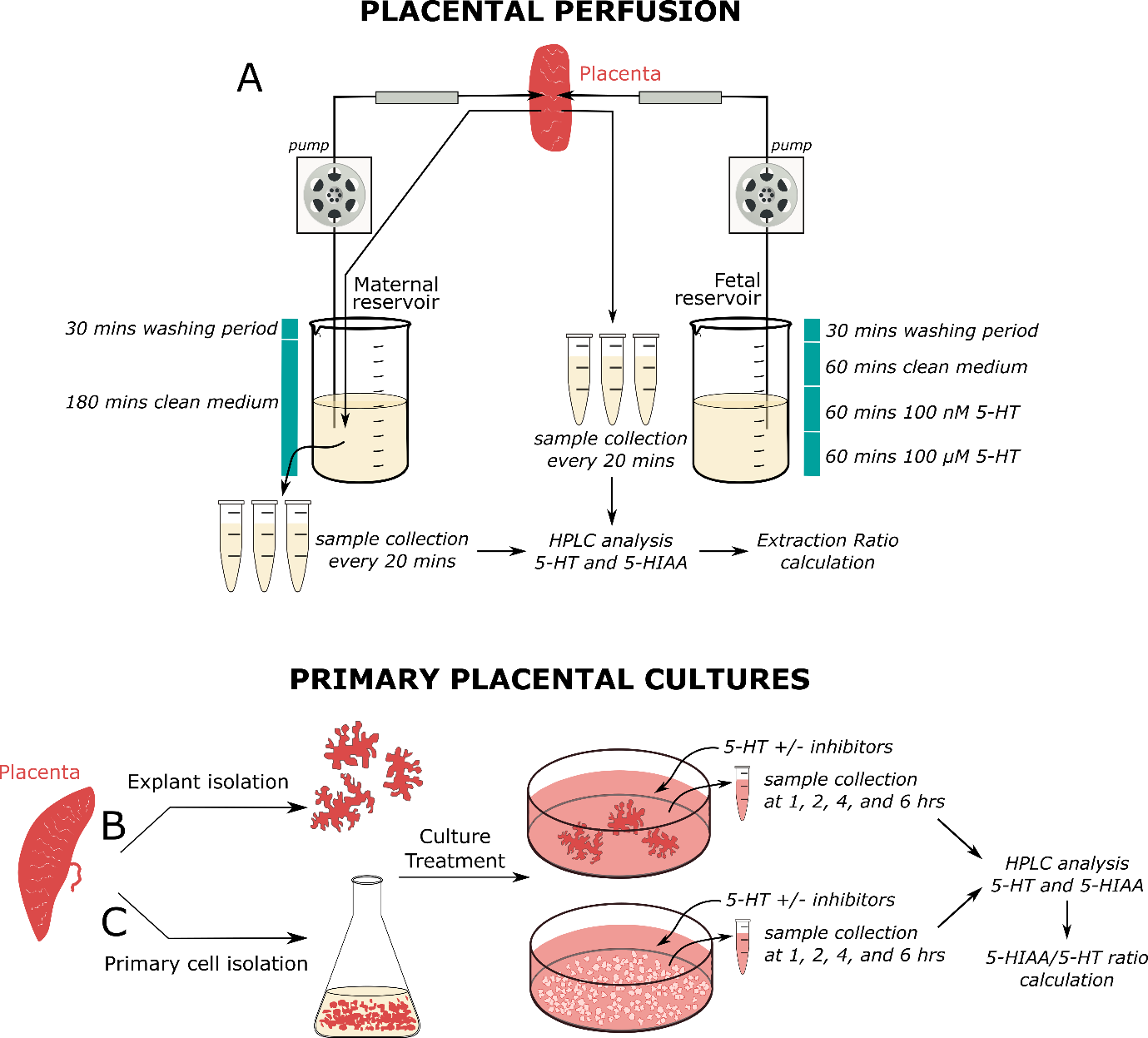


**Figure S1. Schematic representation of the study design.** We employed a systematic approach using advanced organ-, tissue-, and cellular-level models of the human placenta to investigate the transport and metabolism of 5-HT in the fetoplacental unit. (A) Ex vivo perfusion of the human term placenta was performed in a closed system for the maternal circulation (perfusate recirculation); the fetal side was perfused in an open system to ensure steady inflow of 5-HT concentrations (100 nM or 100 µM). The samples were collected from the fetal outflow and maternal reservoir. 5-HT and 5-HIAA levels were analyzed by HPLC, and the placental capacity for removing 5-HT from the fetal circulation was expressed in terms of the extraction ratio. Human placental explants (B) and primary trophoblast cells (C) were isolated from the human term placenta. Functional experiments were performed 72 hours after primary cell isolation, representing the differentiated syncytiotrophoblast and 40 hours after placental explant isolation. Cells/explants were treated with medium supplemented with either 100 nM or 100 µM 5-HT in the absence/presence of various inhibitors for 1, 2, 4, and 6 hours. The cell-free supernatant was used for HPLC measurements, and the results are expressed as the ratio of the 5-HIAA and 5-HT concentrations.


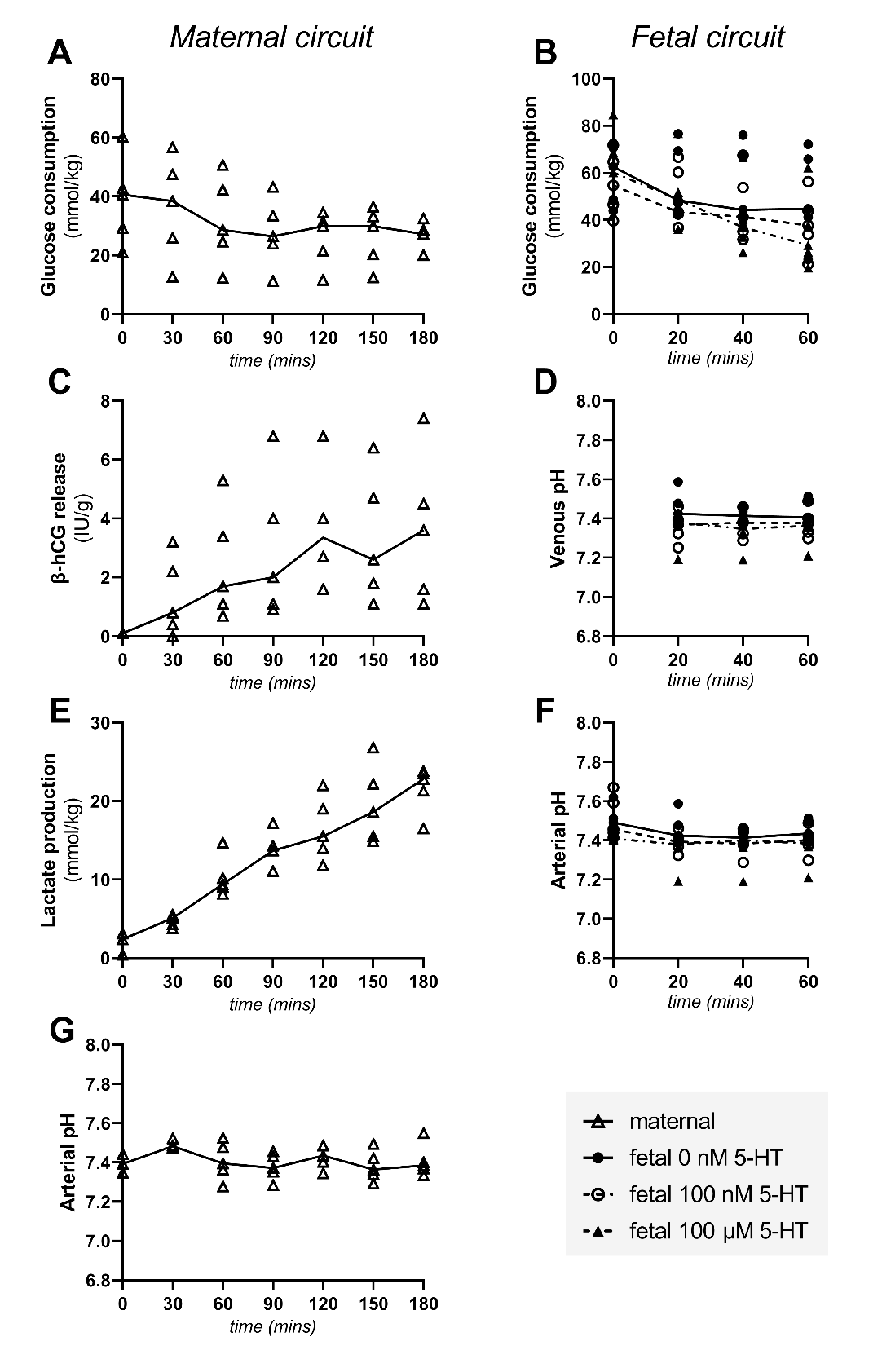


**Figure S2. Quality assessment of placental perfusion experiments.** Parameters monitored during the perfusion included glucose consumption (A, B), lactate production (C), β-hCG release (E), maternal arterial pH (G), and fetal arterial (D) and venous pH (F). Collectively, the metabolic activity and hormone release demonstrates viable placental tissue during the experiment. Data are presented as individual values with the line representing the median; n = 5.


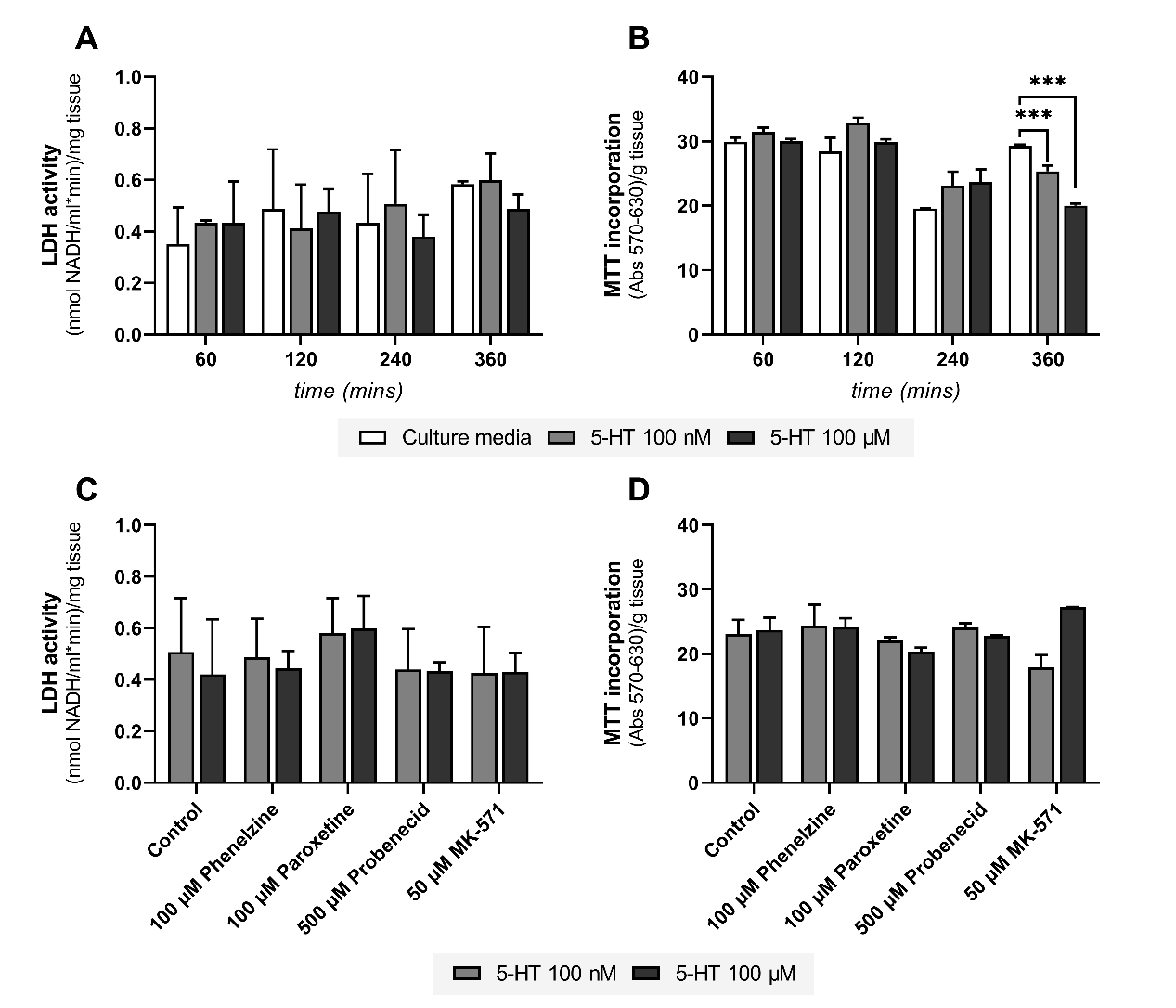


**Figure S3. Viability of human placental explants under different experimental conditions.** LDH activity in the culture media was examined over the course of the experiment (A) and in the presence of inhibitors at 4 hours (C). Additionally, MTT incorporation in the tissue was tested at different times (B) and in the presence of inhibitors at 4 hours (D). Data are shown as median with interquartile range; n ≥ 3. Statistical significance was evaluated using Two-way ANOVA.

**Table S1. Characterization of primary trophoblast cell purity.**

|  | **Median (%)** | **Interquartile range (%)** |
| --- | --- | --- |
| **Cytokeratin-7** | 87.51 | 80.62 - 95.63 |
| **Vimentin** | 10.75 | 4.13 - 15.67 |
| **von Willebrand factor** | 0.16 | 0.05 - 0.76 |

Protein expression of cytokeratin 7, vimentin, and von Willebrand factor, specific markers for epithelial, mesenchymal, and endothelial cells, respectively, was assessed by flow cytometry. The percentage of cells stained for specific proteins was evaluated using the FCS Express Software 7.0.
